# Rapid temporal recalibration to visuo–tactile stimuli

**DOI:** 10.1007/s00221-017-5132-z

**Published:** 2017-11-15

**Authors:** Joachim Lange, Katharina Kapala, Holger Krause, Thomas J. Baumgarten, Alfons Schnitzler

**Affiliations:** Medical Faculty, Institute of Clinical Neuroscience and Medical Psychology, Heinrich Heine University, Düsseldorf, Germany

**Keywords:** Temporal integration, Integration windows, Multisensory, Perceptual cycles, Simultaneity task, Visual

## Abstract

For a comprehensive understanding of the environment, the brain must constantly decide whether the incoming information originates from the same source and needs to be integrated into a coherent percept. This integration process is believed to be mediated by temporal integration windows. If presented with temporally asynchronous stimuli for a few minutes, the brain adapts to this new temporal relation by recalibrating the temporal integration windows. Such recalibration can occur even more rapidly after exposure to just a single trial of asynchronous stimulation. While rapid recalibration has been demonstrated for audio–visual stimuli, evidence for rapid recalibration of visuo–tactile stimuli is lacking. Here, we investigated rapid recalibration in the visuo–tactile domain. Subjects received visual and tactile stimuli with different stimulus onset asynchronies (SOA) and were asked to report whether the visuo–tactile stimuli were presented simultaneously. Our results demonstrate visuo–tactile rapid recalibration by revealing that subjects’ simultaneity reports were modulated by the temporal order of stimulation in the preceding trial. This rapid recalibration effect, however, was only significant if the SOA in the preceding trial was smaller than 100 ms, while rapid recalibration could not be demonstrated for SOAs larger than 100 ms. Since rapid recalibration in the audio–visual domain has been demonstrated for SOAs larger than 100 ms, we propose that visuo–tactile recalibration works at shorter SOAs, and thus faster time scales than audio–visual rapid recalibration.

**Electronic supplementary material:**

The online version of this article (10.1007/s00221-017-5132-z) contains supplementary material, which is available to authorized users.

## Introduction

Our sensory systems permanently receive multiple bits of information from complex natural events. Usually, this information is multimodal and thus the input is initially processed in different sensory systems. To generate a coherent perception of our environment, the brain must constantly decide whether the input comes from the same source and consequently need to be integrated to a single perceptual event. Alternatively, if the information originates from multiple, different sources, the sensory input needs to be segregated into multiple separate perceptual events.

One major determinant for integration or segregation is temporal proximity between the multiple sensory inputs. Stimuli that occur in close temporal proximity are more likely to originate from one source and thus are more likely to produce enhanced neural activity (Stein and Wallace 1996; Kayser and Logothetis 2007). Also, on a perceptual level, temporal proximity is an important determinant for integration. For example, when congruent lip movements and auditory speech reach the brain within a short time window of a few milliseconds, they are more likely to originate from the same source than lip movements and auditory speech separated by seconds. Thus, lip movements and speech signals in close temporal proximity are more likely to be perceived as one congruent speech event (van Wassenhove et al. 2007). The brain can also use information about temporal structure or rhythmicity as predictive cues for sensory integration (Luo and Poeppel 2007; Vroomen and Stekelenburg 2010). Such findings led to the hypothesis of temporal integration windows for multisensory perception (Pöppel 1997; van Wassenhove et al. 2007). If multiple stimuli fall within an integration window, they will be integrated and lead to a coherent perception of a single event (van Wassenhove et al. 2007; Cecere et al. 2015; Baumgarten et al. 2015; VanRullen 2016). Otherwise, the two stimuli will be processed and subsequently perceived as two separate events.

In natural events, multisensory stimuli often exhibit stimulus onset asynchronies (SOA), which can depend on several factors such as distance of the source, neural transduction latencies or neural processing times (Alais and Carlile 2005; Harrar and Harris 2005). It has been shown that the brain has developed mechanisms to adapt to such SOAs and to compensate for the variability of temporal information (Harrar and Harris 2005; Thorne et al. 2011). Such adaptation can occur within minutes. For example, in simultaneity judgments, the brain adapts to constant exposure to asynchronous stimuli after a few minutes. This adaptation mechanism shifts the point of subjective simultaneity (PSS) towards the modality that was the leading modality during the adaption phase. Such ‘recalibration’ of (learned) temporal dependencies has been repeatedly shown for a variety of multisensory stimuli (Fujisaki et al. 2004; Hanson et al. 2008; Harrar and Harris 2008).

Recently, van der Burg et al. (2013) demonstrated that recalibration can occur even more rapidly in the absence of a prolonged adaptation phase. In an audio–visual simultaneity task, the PSS was found to be quickly recalibrated by the modality order in the preceding trial. Such rapid recalibration in only a single trial, however, could only be shown for audio–visual simultaneity judgments, while no rapid recalibration effects were found in visuo–tactile and audio–tactile simultaneity judgments tasks (van der Burg et al. 2015b).

Van den Burg et al. (2015b) concluded that in contrast to long-term adaptation, rapid recalibration is unique to audio–visual stimuli. However, an alternative explanation for the uniqueness of audio–visual stimuli might be the temporal scale on which multisensory stimuli are processed. In their study, van den Burg et al. used SOAs of ≥ 100 ms. Such SOAs are well in the range of proposed temporal integration windows for visual or audio–visual integration (van Wassenhove et al. 2007; Romei et al. 2012; Cecere et al. 2015). Integration windows for tactile stimuli, however, have been shown to act on shorter time scales of ~ 50 ms (Baumgarten et al. 2015, 2017). Thus, temporal integration windows for other multisensory stimuli involving tactile stimuli might also act on shorter time scales (Harrar and Harris 2005; Gick et al. 2010).

In this study, we investigated rapid recalibration for visuo–tactile stimuli. We hypothesized that rapid recalibration of perception for visuo–tactile stimuli might act on shorter time scales compared to audio–visual stimuli. To this end, we adapted the paradigm of van der Burgh et al. (2015b), however, using also visuo–tactile stimuli with shorter SOAs. Our results demonstrate that rapid recalibration also occurs for visuo–tactile stimuli. The recalibration, however, was only found for short SOAs in the preceding, recalibrating trial.

## Methods

### Participants

Twenty-two subjects [six male, age: 24.6 ± 3.0 years (mean ± SD)] participated in this study after giving written informed consent in accordance to the declaration of Helsinki and the Ethical Committee of the Medical Faculty, Heinrich Heine University Düsseldorf. All participants had normal or corrected-to-normal vision and reported no somatosensory deficits or known history of neurological disorders.

Four subjects had to be excluded from analysis due to implausible response patterns during the task (see below) so that finally 18 subjects [six male, age 24.4 ± 3.3 years (mean ± SD)] were included in the analyses.

### Stimuli and paradigm

Subjects were sitting comfortably in a sound-attenuated room with dimmed light. Subjects’ head was placed in a helmet-shaped inlay of a magnetoencephalograph (MEG). While we recorded neuronal activity with the MEG simultaneously to the task, in this study we will focus solely on the behavioral parameters of the task.

**Fig. 1:**
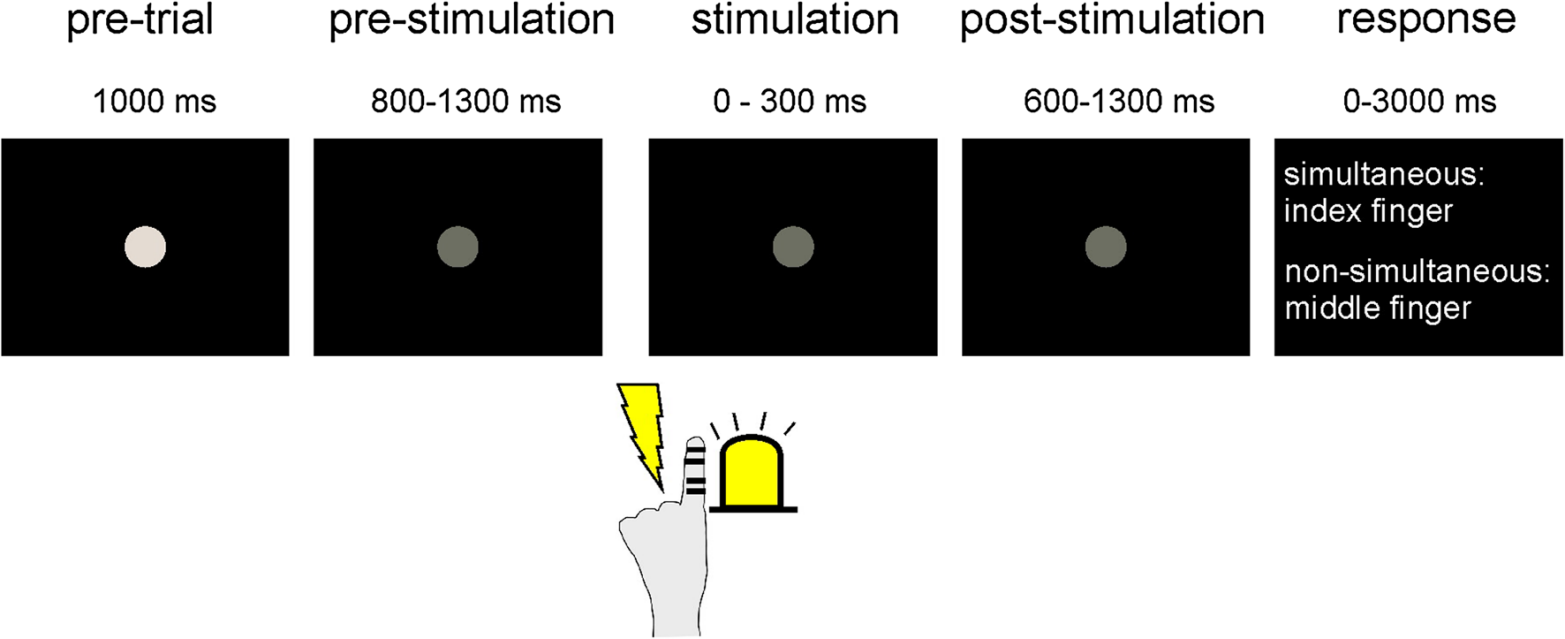
Experimental setup. Subjects fixated a central fixation dot. After a jittered period, they received one electro-tactile stimulus on their left index finger and one visual stimulus via an LED attached to the left index finger. Both stimuli were presented in varying order and with varying stimulus onset asynchronies (SOA). After another jittered period, subjects reported via button press with their right hand whether the visual and tactile stimuli were perceived as simultaneous or non-simultaneous. Button configuration was randomized from trial to trial

Subjects fixated a central grey dot presented via a projector (PT-D7700E, Panasonic, Japan) located outside the magnetically shielded room on a screen 57 cm in front of them. After 1000 ms the dot decreased in luminance indicating the start of the stimulation period (Fig. 1). After a jittered period of 800–1300 ms subjects received visuo–tactile stimulation. Electro-tactile stimulation was generated by a Stimulus Current Generator (DeMeTec GmbH, Germany) and applied by electrical pulses (duration 0.3 ms) via ring-electrodes attached to the tip of the left index finger. Stimulation amplitudes (2.8 ± 0.9 mA) were individually adjusted prior to the experiment to a level where subjects could clearly perceive stimulation, but below the pain threshold. Visual stimulation was applied via a light emitting diode (LED) attached to the tip of the left index finger, just below the electrodes for electrical stimulation. Stimulation intensity was set that the light was clearly visible and intensity was kept constant for all subjects (duration 5 ms). The left hand was placed comfortably on a table so that stimulation appeared at ~ 20° left and below the fixation dot. In each trial, one visual and one tactile stimulus were applied with varying stimulus onset asynchronies (SOAs) of ± 300, ± 150, ± 125, ± 100, ± 75, ± 50, ± 25 or 0 ms, with negative values indicating visual stimulation occurring before tactile stimulation and vice versa. SOAs with ± 300, ± 150 and 0 ms were presented in ten trials, the remaining SOAs were presented in 50 trials in pseudo-randomized order, each. Stimulation was followed by another jittered time period (600–1300 ms) during which only the fixation dot was visible, before response instructions were presented on the screen. In a forced-choice paradigm, subjects had to report whether they perceived the visuo–tactile stimulation as simultaneous or non-simultaneous by pressing respective buttons with the index and middle finger of their right hand. Button configurations were randomized from trial to trial to minimize response preparation. If subjects did not respond within 3000 ms after or before the response instructions were presented, a warning message was presented on the screen and the trial was repeated at the end. All in all, 550 trials were presented with self-paced breaks after every 150 trials.

The task was preceded by a training phase of ~ 5 min to familiarize subjects with the task. Prior to the experiment, general instructions were visually presented on the screen. Subjects received no feedback on their responses and remained naïve towards the aim of the task.

### Analysis of behavioral data, fitting procedure and statistical analysis

To analyze whether the subjective perception in one trial was influenced by the stimulation in the preceding trial, we separated trials according to the stimulation in the preceding trial. That is, one condition contained all trials for which the stimulation order of the preceding trial was tactile–visual (i.e., all trials with preceding trial having a positive SOAs, denoted tv) while one condition contained all trials for which the stimulation order in the preceding trial was visual–tactile (i.e., all trials with preceding trial having a negative SOAs, named vt). For the SOAs with ± 300, ± 150 and 0 ms, this resulted in 4.8 ± 1.2 trials (mean ± SD across subjects and SOAs, range 3.4–6.1) for the tv conditions and 5.1 ± 1.2 trials (mean ± SD, range 3.9–6.1) for the vt conditions. For the remaining SOAs, this resulted in 24.5 ± 1.7 trials (mean ± SD, range 21.5–26.6) for the tv conditions and 24.2 ± 1.9 trials (mean ± SD, range 21.7–27.6) trials for the vt conditions. For each condition, SOA and subject, we computed mean response rates by averaging the simultaneous reports across trials. Finally, we averaged mean response rates per condition and SOA across subjects.

Four subjects had to be excluded from analyses, because they showed an implausible response pattern, e.g., response distributions showing not the expected bell-like shape (indicating excessive guessing or not understanding the task), unusual high number of simultaneity reports for long SOAs (>25% simultaneity reports, indicating strong bias towards reporting “simultaneous”), or unusual low number of simultaneity reports for short SOAs (<75% simultaneity reports, indicating strong bias towards reporting “non-simultaneous”, Fig. S1).

**Fig. 2:**
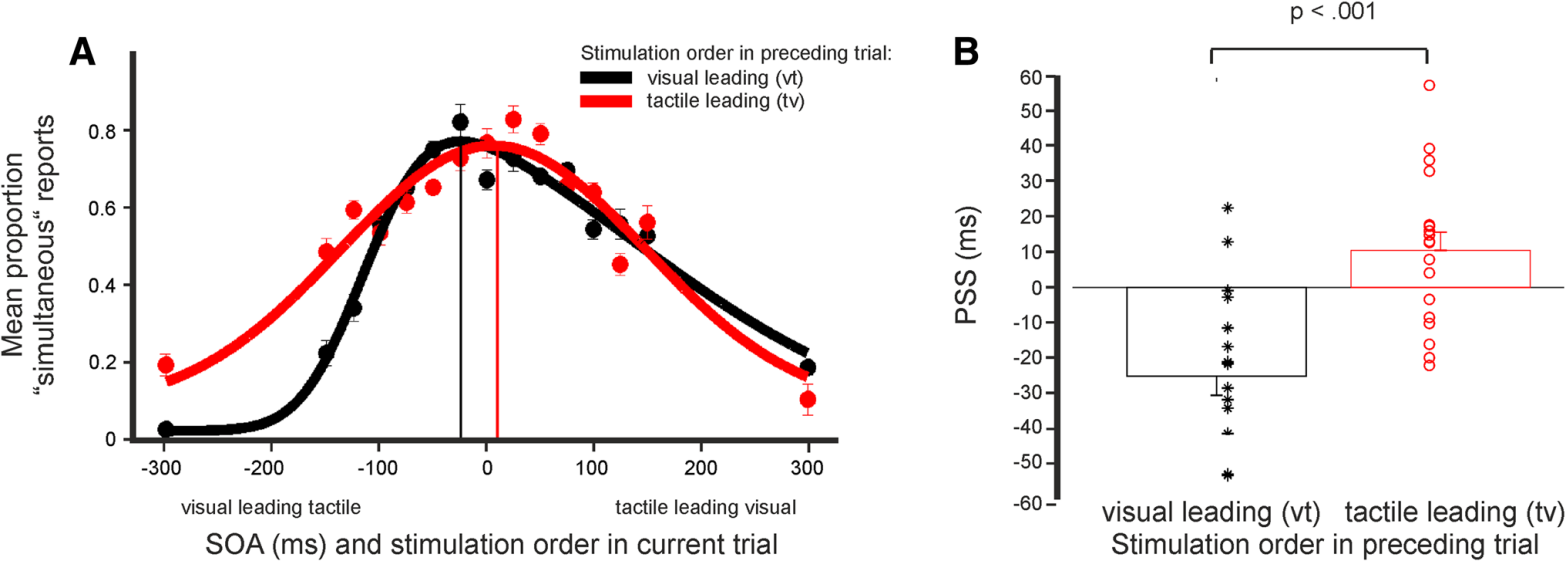
**A** Mean proportion of simultaneity reports as a function of SOA. Trials were split depending on the stimulation order in the preceding trial (visual–tactile or tactile–visual). Red dots represent subjects’ responses (mean ± SEM) if the stimulation order in the preceding trial was tactile–visual. Black dots represent responses if the stimulation order in the preceding trial was visual–tactile. Red and black curves show the best fitting skewed normal distribution fitted to the averaged data. Red and black vertical lines indicate the respective PSS (point of subjective simultaneity; i.e., the maximum of the fitted function). **B** PSS were determined for each subject and then averaged across subjects. Black stars and red dots represent PSS of individual subjects, the bars represent mean ± SEM across subjects. Black and red bars (and stars and dots, respectively) represent PSS if the stimulation order in the preceding trial was visual–tactile or tactile, visual, respectively

The point of subjective simultaneity (PSS) denotes the SOA, at which subjects most likely report the stimulation as simultaneous. To estimate the PSS, we modelled each subject’s response distribution. Since response distributions showed an asymmetrical pattern (Figs. 2, S1), we used skewed Gaussian-like functions (Yarrow et al. 2011; van der Burg et al. 2015b):

$${\text{SR}}\left( {{\text{SOA}}} \right)={\text{norm}}\left( {{\text{SOA}}} \right) \times e \times {\text{cdf}}\left( {{\text{SOA}}} \right)$$

with

$${\text{norm}}=a+~\frac{1}{b} \times \exp \left( {\frac{{ - {{\left( {{\text{SOA}} - c} \right)}^2}}}{{{d^2}}}} \right)$$

denoting the normally shaped distribution, cdf the cumulative density function of the normal function, *a, b, c, d, e* parameters to be fitted, and SR the simultaneity reports as a function of the SOA.

We estimated the PSS for trials in the vt and tv conditions (PSS_vt_ and PSS_tv_) for each subject separately. In addition, we estimated the PSS including all trials, i.e., before considering putative rapid recalibration effects. Finally, we statistically compared PSS_vt_ and PSS_tv_ across subjects by means of a two-tailed paired samples *t* test (after confirming that the distribution of individual PSS did not significantly differ from a normal distribution by means of a Kolmogorov–Smirnov-test).

PSS_vt_ and PSS_tv_ might not only be influenced by the stimulation order in the preceding trial (i.e., visual–tactile vs. tactile–visual), but also by the length of the SOA in the preceding trial. Therefore, we additionally split trials in conditions with the preceding trial having tactile–visual stimulation and an SOA ≥ 100 ms (denoted tv_long) and in conditions with the preceding trial having tactile–visual stimulation and an SOA < 100 ms (named tv_short). The same analysis was performed when preceding trials had visual–tactile stimulation (vt_long and vt_short). After confirming that the distribution of individual PSS did not significantly differ from a normal distribution by means of a Kolmogorov–Smirnov-test, the four resulting PSS were statistically compared by a 2 × 2 repeated measures ANOVA with factors stimulation order (tv or vt) and SOA duration (short or long), followed by post-hoc paired sample *t* tests.

Finally, we computed effect sizes (Cohen’s *d*) according to the formula

$$d=~\frac{{{M_1} - {M_2}}}{{\sqrt {\frac{{S_{1}^{2}+~S_{2}^{2}}}{2}} }}$$

with M1 and M2 denoting the means and S1 and S2 denoting the standard deviations of the two compared conditions.

All analyses were performed using Matlab (The Mathworks Inc., Natick/MA, USA).

## Results

### Behavioral results

Subjects received one visual and one tactile stimulus in varying order and with varying stimulus onset asynchronies (SOA) and had to report whether they perceived the two stimuli as simultaneous or non-simultaneous. We investigated whether the subjective perception of simultaneity depended on the stimulation pattern in the preceding trial, i.e., whether subjects show rapid recalibration in a visuo–tactile task. To this end, we separated trials in conditions depending on stimulation order in the preceding trial (visual leading tactile, denoted vt, or tactile leading visual, denoted tv).

For both conditions, subjects showed the lowest number of simultaneity reports for the largest SOAs (± 300 ms) and a peak of simultaneity reports at the point of subjective simultaneity (PSS, Fig. 2A). To determine the PSS, we fitted skewed Gaussian function to each distribution. PSS_vt_ will denote the PSS for trials in which the stimulation order in the preceding trial was visual–tactile; and PSS_tv_ for stimulation order tactile–visual in preceding trials. In addition, we determined the PSS for the base simultaneity reports, i.e., including all trials irrespective of the stimulation order in the preceding trial.

We found that the modality order of the stimulation in the preceding trial influenced subjects’ perception of simultaneity in the current trial. When we fitted the mean simultaneity responses averaged across subjects, the PSS_vt_ was − 29.8 ms and the PSS_tv_ 4.3 ms (Fig. 2A). When we fitted the mean simultaneity reports including all trials irrespective of stimulation order in the preceding trial, the PSS was 3.9 ms (Fig. S1A).

Additionally, we determined individual PSS by fitting each subject’s response distributions. Averaged across subjects, PSS_vt_ were − 25.1 ± 5.5 ms (mean ± SEM) and PSS_tv_ were 10.5 ± 5.1 ms (Fig. 2B). Statistical analysis revealed a highly significant difference between conditions PSS_vt_ and PSS_tv_ [*t*(17) = 4.971; *p* < .001). The effect size was *d* = 1.584.

Next, we investigated whether the PSS additionally depended on the length of the SOA in the preceding trial. Therefore, we additionally split the conditions in “long” (> 100 ms or < − 100 ms, respectively) and “short” SOAs (< 100 ms or > − 100 ms, respectively). When we fitted the mean simultaneity responses averaged across subjects, the PSS_vt_long_ was 12.8 ms and the PSS_vt_short_ was − 25.7 ms. The PSS_tv_long_ was − 0.8 ms and the PSS_tv_short_ was 39.5 ms.

**Fig. 3:**
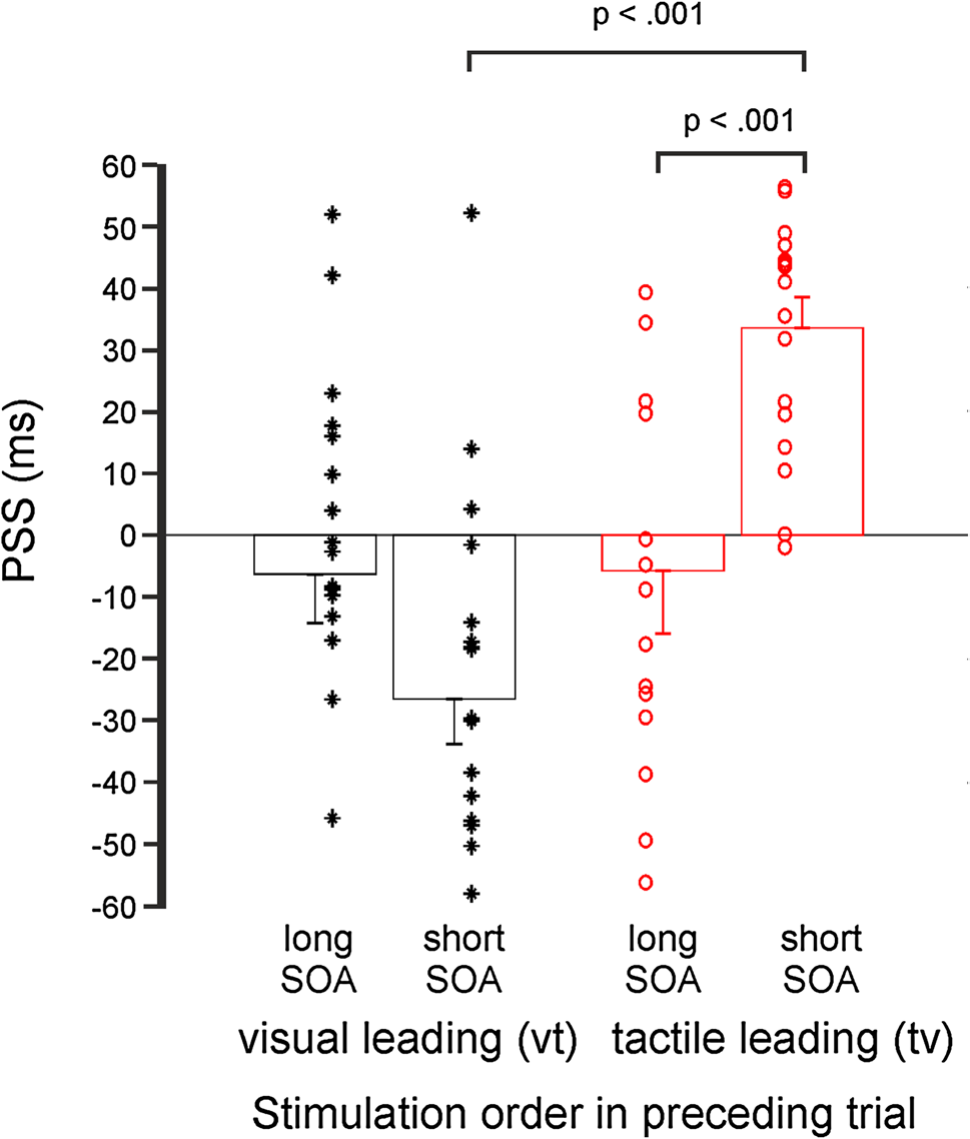
Same as Fig. 2B, but now trials were additionally split into conditions depending on the SOA in the preceding trial (long SOA indicating length of SOAs ≥ 100 ms; short SOA indicating length of SOAs < 100 ms)

Additionally, we determined individual PSS by fitting each subjects’ response distributions. Averaged across subjects, PSS_vt_long_ were − 6.4 ± 7.9 ms (mean ± SEM) and PSS_vt_short_ were − 26.4 ± 7.2 ms. The averaged PSSt_v_long_ was − 5.8 ± 10.1 ms (mean ± SEM) and the averaged PSS_tv_short_ was 32.5 ± 4.6 ms (Fig. 3).

A 2 × 2 ANOVA with factors modality order in the preceding trial (vt or tv) and SOA duration (long or short) revealed a highly significant main effect for order [*F*(1,53) = 28.280; *p* < .001] and a highly significant interaction effect [*F*(1,53) = 19.546; *p* < .001], while the main effect of duration was not significant [*F*(1,53) = 1.064; *p* = .317].

Post-hoc *t* tests revealed highly significant differences between the PSS_tv_short_ and PSS_tv_long_ [*t*(17) = 4.353; *p* < .001; d = 1.152] and between PSS_tv_short_ and PSS_vt_short_ [*t*(17) = 8.104; *p* < .001; *d* = 2.292]. The other comparisons did not reach statistical significance (*p* ≥ .136; *d* ≤ 0.626).

## Discussion

After an adaptation phase of sustained repetitive stimulation with asynchronous multisensory stimuli, the brain adapts by reducing the perceived stimulus onset synchrony (SOA) between the two asynchronous stimuli. This so-called temporal recalibration has been found for audio–visual, audio–tactile, and visuo–tactile stimuli (Fujisaki et al. 2004; Hanson et al. 2008). Recently, studies have shown that temporal recalibration can be induced even by a single trial, the so-called rapid recalibration (van der Burg et al. 2013, 2015b). Unlike recalibration following sustained adaptation, rapid recalibration has been reported only for audio–visual stimuli, while it was absent for visuo–tactile stimuli (van der Burg et al. 2015b; Alais et al. 2017). In the present study, however, we were able to demonstrate rapid recalibration for visuo–tactile stimuli. A detailed analysis revealed that significant rapid recalibration occurred most prominently if the SOA in the preceding, recalibrating trial was lower than 100 ms. In contrast, we could not find visuo–tactile rapid recalibration effects for SOAs ≥ 100 ms.

We found that visuo–tactile and tactile–visual stimulation had different effects on perception of the subsequent trials. When we included all trials in the analyses, i.e., before considering putative rapid recalibration effects, the point of subjective simultaneity (PSS) was 3.9 ms. While visuo–tactile in the preceding trial shifted the PSS towards negative SOAs (− 29.8 ms), tactile–visual stimulation in the preceding trial had virtually no effect on the PSS (4.3 ms). Only when we additionally analyzed only tactile–visual stimulation with short SOAs (< 100 ms) in the preceding trial, the PSS was shifted towards positive SOAs (39.5 ms). The different effects of visual–tactile and tactile–visual stimulation on simultaneity reports might be due to different mechanisms underlying visuo–tactile and tactile–visual integration. That is, the order of the stimulation might have an influence on integration processes. For example, a recent study reported that audiovisual temporal integration was different for auditory-leading vs. visual-leading stimulus pairs (Cecere et al. 2016). The reason for the difference might that audio–visual and visuo-auditory integration engages different neuronal mechanisms possibly due to different multisensory sampling mechanisms depending on leading sense (Cecere et al. 2017). Similar differences for visuo–tactile and tactile–visual stimulation might be responsible for the differences we found in the present study.

Previous studies reported rapid recalibration effects for audio–visual stimuli, but were unable to demonstrate rapid recalibration for visuo–tactile stimuli (van der Burg et al. 2015b; Alais et al. 2017). A major difference between our study and previous studies is the length of the SOAs between visual and tactile stimuli. Typically, previous studies used SOAs with a minimum length of 90–100 ms (Hanson et al. 2008; Harrar and Harris 2008; van der Burg et al. 2013, 2015b; Alais et al. 2017). Such a long SOA was sufficient to induce recalibration effects after sustained adaptation phases for audio–visual, audio–tactile and visuo–tactile stimuli. In addition, SOAs ≥ 100 ms have been shown to induce rapid recalibration for audio–visual stimuli but not for visuo–tactile stimuli (van der Burg et al. 2015b; Alais et al. 2017). Our results confirm these previous results. When we analyzed only those trials for which the preceding trial had an SOA of minimum 100 ms, we were unable to find significant rapid recalibration effects for our visuo–tactile stimuli. With smaller SOAs (< 100 ms), however, we could demonstrate rapid recalibration effects. We thus propose that rapid recalibration of visuo–tactile stimuli works on a shorter time scale (i.e., < 100 ms) than recalibration after a sustained adaptation phase (with SOAs ≥ 100 ms). In addition, we propose that rapid recalibration of visuo–tactile stimuli works on a different time scale (i.e., < 100 ms) than rapid recalibration of audio–visual stimuli (≥ 100 ms).

The length of the SOA is the major difference between our study and previous studies. Yet, there are a couple of additional differences that might affect rapid recalibration effects. While previous studies mostly used tactile stimulators that induced pressure to the finger, we used electrical stimulations. These two stimulation techniques might stimulate different receptors of the skin and thus might contribute differently to temporal tactile perception (Kandel et al. 2000). In addition, we used shorter stimulus durations than previous studies (~ 50 ms in previous studies, ≤ 5 ms in our study), which might have influenced simultaneity judgements (Boenke et al. 2009; Stevenson and Wallace 2013). Finally, we presented visual and tactile stimuli spatially aligned in the periphery, while others presented stimuli at different locations or centrally. While spatial congruency has been shown to be irrelevant for rapid recalibration in the audio–visual domain and for visuo–tactile recalibration after sustained adaptation (Keetels and Vroomen 2007; Ho et al. 2015), it cannot be excluded that for rapid recalibration spatial congruency is necessary. Yet, despite these differences between studies, we were unable to show rapid recalibration effects for SOAs larger than 100 ms. Therefore, we believe that a potential influence of any of these factors on rapid recalibration of visuo–tactile stimuli should be either negligible or only present for SOAs smaller than 100 ms. In conclusion, we propose that rapid recalibration depends predominantly on the SOA of the preceding trial. We could show that the critical SOA for rapid recalibration for visuo–tactile stimuli is < 100 ms, while the critical SOA is 100 ms (and potentially longer) for audio–visual stimuli (van der Burg et al. 2013, 2015b).

This new finding of rapid visuo–tactile recalibration raises two questions. First, why does rapid recalibration of audio–visual and visuo–tactile stimuli occur on different time scales? In addition, previous studies have shown that recalibration after a sustained adaptation phase with SOAs of 100 ms can induce adaptation phases for both, audio–visual and visuo–tactile stimuli. Therefore, the second question is why rapid and sustained recalibration of visuo–tactile stimuli occurs at different time scales?

For audio–visual stimuli, sustained adaptation produces a sustained recalibration effect that lasts up to a few minutes. Rapid recalibration, however, produces transient effects that change from trial to trial (Van der Burg et al. 2015a). Both effects have been found to occur in parallel in one experiment, but independently from each other. Thus, it has been suggested that both effects occur on separate and independent time scales. Yarrow et al. (2011) suggested that rapid and sustained recalibration reflect different level of a sensory-decisional process. Sustained recalibration may be caused by criterion shifts in higher level decisional process and thus might be considered as a supramodal effect that should affect audio–visual, visuo-tactile, and audio–tactile stimuli in a similar way (Yarrow et al. 2011). Indeed, it has been shown that audio–visual, visuo-tactile, and audio–tactile stimuli show sustained recalibration effects of comparable size (Fujisaki et al. 2004; Keetels and Vroomen 2007; Hanson et al. 2008). In contrast, rapid recalibration has been suggested to reflect shifts in temporal alignment in sensory processes (Kösem and van Wassenhove 2012).

A potential reason for the differences between audio–visual and visuo–tactile rapid recalibration might thus be found in the different temporal processing and integration of these stimuli. For example, temporal integration windows for visuo–tactile stimuli have been found to be smaller than for audio–visual stimuli (Harrar and Harris 2008; Fujisaki and Nishida 2009; Noel et al. 2015). It has been argued that audio–visual integration needs flexible integration mechanisms. For example, integration of lip movements and voices typically improves speech processing and perception. It would be beneficial if such integration mechanisms rapidly adapt to inter-individual differences, intra-individual changes of speed or rhythmicity, or changes of the distance between source and observer. In contrast to auditory signals, the distance between the source of touch and the observer cannot change significantly as touch is always applied to the skin. The major source of variability in visuo–tactile integration comes from different neural transduction times for touch at different body parts. Typically, the differences of transduction times are less than 100 ms (Harrar and Harris 2008). From this point of view, one might expect that visuo–tactile integration time windows should rapidly and flexibly adapt at time scales below 100 ms. Visuo–tactile recalibration might thus work on a different, shorter timescale than audio–visual rapid recalibration.

Due to the inter- and intra-individual factors, audio–visual integration needs to adapt to higher variability than visuo–tactile integration. We might speculate that due to this higher variability, audio–visual stimuli need longer integration windows, which might rely more strongly on the temporal resolution of the visual modality. On the other hand, visuo–tactile stimuli show less variation as the tactile source shows less variation (e.g., each location on body has fixed latency of neural transduction). Therefore, visuo–tactile stimuli need shorter integration windows, which additionally might rely more strongly on the temporal processing of tactile stimuli. The contribution of visual and tactile information on multisensory processing might vary and rely on reliability of the sources (Ernst and Banks 2002; Ernst and Bülthoff 2004).

Recent studies revealed that cycles of neuronal oscillations form the neuronal basis of these integration windows (Cecere et al. 2015; Baumgarten et al. 2015, 2017; VanRullen 2016). Different frequency bands seem to play a different role for temporal perception in different modalities, with the theta- (~ 4–7 Hz) and alpha- (~ 8–12 Hz) band playing a dominant role in the visual and audio–visual domain (Romei et al. 2012; Landau and Fries 2012; Cecere et al. 2015; VanRullen 2016), while the beta-band (~ 13–25 Hz) plays a dominant role in the somatosensory domain (Baumgarten et al. 2015, 2017). We might speculate that repetitive or even single stimulation triggers these frequencies relevant for temporal perception. Adaptation to the preceding trial(s) might be reflected by adaptation of the carrier frequency to slightly lower or higher frequencies, respectively. In this framework, adaptation would take place most prominently if the adaption stimulus is able to trigger the frequency relevant for temporal perception. Since the frequencies for temporal perception differ between visual and tactile modalities, we would expect different SOAs to trigger adaptation effects. Since in the (audio-)visual modality alpha frequencies with cycle lengths of ~ 100 ms seem to be relevant, we would expect maximal adaptation and recalibration effects in the visual modality with SOAs of ≥ 100 ms as found in van der Burg et al. (2013, 2015b). In contrast, we would expect maximal adaptation and recalibration effects in the somatosensory domain for SOAs corresponding to the beta-band (~ 13–25 Hz), i.e., for SOAs of ~ 40–80 ms, but lesser or even absent recalibration effects for SOAs ≥ 100 ms. The results of the present study are in line with the proposed framework as we found rapid visuo–tactile recalibration effects for SOAs < 100 ms, but not for SOAs ≥ 100 ms, while other studies found rapid audio–visual recalibration for SOAs ≥ 100 ms (van der Burg et al. 2013, 2015b).

In summary, we provide evidence for rapid recalibration in the visuo–tactile domain. The rapid recalibration, however, could only be demonstrated if the SOA in the preceding trial was smaller than 100 ms. Since rapid recalibration in the audio–visual domain has been demonstrated for SOAs ≥ 100 ms, we propose that visuo–tactile and audio–visual rapid recalibration work on different time scales. We suggest that the neural basis for these differences might be found in different frequency bands relevant for audio–visual and (visuo-) tactile temporal perception.

## Electronic supplementary material

Below is the link to the electronic supplementary material.

Supplementary Figure S1: Behavioral results without considering rapid recalibration. A) Proportion of simultaneity reports as a function of SOA, averaged across subjects. The black curve shows the best fitting skewed normal distribution fitted to the averaged data. The black vertical lines indicate the respective PSS (point of subjective simultaneity; i.e., the maximum of the fitted function). B) Same as A) but now showing the individual behavioral results for all 18 subjects included in A) and in the further analyses. C) Same as B) but now showing the four subjects excluded from further analyses either due to a strong bias towards reporting “non-simultaneous” (< 75% simultaneity reports for short SOAs, figures 1-3) or due to a strong bias towards reporting “simultaneous” (> 25% simultaneity reports for long SOAs, figure 4) (TIF 333 KB)
